# Equivalent Improvements in Sleep Duration and Sleep Quality Regardless of Program Delivery Modality: The SLeep Education for Everyone Program (SLEEP)

**DOI:** 10.3390/clockssleep5020018

**Published:** 2023-04-18

**Authors:** Dawn A. Contreras, Elizabeth Williams, Robin M. Tucker

**Affiliations:** 1 Michigan State University Extension, East Lansing, MI 48824, USA; contrer7@msu.edu (D.A.C.); josaitis@msu.edu (E.W.); 2 Department of Food Science and Human Nutrition, Michigan State University, East Lansing, MI 48824, USA

**Keywords:** sleep education, sleep hygiene, insomnia, sleep health

## Abstract

Sleep issues are pervasive, and treatment can be difficult to access, if available at all. The purpose of this study was to test whether the delivery modality (online vs. in person) of the SLeep Education for Everyone Program (SLEEP) influenced programmatic outcomes. A total of 60 participants completed the study, 28 in the online group and 32 in the in-person group. Across all participants, SLEEP improved sleep duration, sleep quality, and sleep hygiene behaviors (*p* < 0.001 for all). When comparing delivery modality, sleep duration and quality improved similarly between groups; however, sleep hygiene behaviors improved more in the in-person group (*p* = 0.033). Sleep hygiene scores did not correlate with sleep duration or quality after the program. Based on these findings, SLEEP appears to be equally effective in improving sleep duration and quality when delivered online or in person. These findings suggest that SLEEP can be delivered based on the organization’s and participant’s resources, needs, and preferred style of interaction.

## 1. Introduction

Sleep issues, including insufficient and poor-quality sleep, are a global concern [1,2]. Recent data from multiple countries suggests sleep outcomes such as difficulty falling asleep, trouble staying asleep, and sleep duration were worsening prior to the COVID-19 pandemic [3,4,5]. Further, several studies reported the pandemic was detrimental to sleep in multiple countries [6,7,8,9], with approximately 40% of the individuals surveyed across 13 countries reporting sleep issues [9]. While there is considerable variability in the prevalence of sleep problems at the country level, e.g., [2,10], various single-country studies prior to the pandemic are consistent with this ~40% figure, including the U.S. (in 2018, 35.6% reported short sleep) [4] and Finland (occasional insomnia reported by 44.8% of respondents in 2013) [5]. Regardless of the exact figures, clearly, many people struggle with sleep issues.

In the U.S., access to sleep health care, particularly for those who most need it, can be challenging [11]. Prohibitive cost, as well as lack of time, provider availability, and transportation to appointments, can make treatment of clinical and subclinical sleep disorders out of reach for many individuals [12]. The SLeep Education for Everyone Program (SLEEP) was designed as a lay healthcare provider-delivered online intervention to address these gaps in sleep health care.

SLEEP consists of six sessions designed to improve sleep-related knowledge and behaviors in an adult population. SLEEP was developed by sleep researchers, a sleep medicine certified practitioner, and health educators from Michigan State University Cooperative Extension (MSUE), as well as current and former Extension programming participants. SLEEP is currently delivered free of charge by MSUE. Session topics include sleep hygiene training, Stimulus Control Therapy, relaxation techniques, physical activity, and goal setting to increase sleep duration and quality. Each session includes a short video covering the topic of the week, a group discussion of the topic, and a goal-setting activity. SLEEP has been shown to significantly improve sleep quality, sleep hygiene behaviors, and daytime sleepiness in an intervention group of older adults compared to the control group [13]. Those improvements persisted for at least six months after the program ended [14].

SLEEP was initially developed for online delivery to maximize programmatic reach and to respond to pandemic-related distancing guidelines; its effectiveness when presented in person was unknown. The U.S. state of Michigan, where SLEEP was conceived, spans over 250,000 km^2^ (96,000 mi^2^) and consists of large urban and small rural areas that are separated by a considerable distance. The vast distances mean that MSUE educators are unable to provide programming in every location in the state. Online delivery of programming reduces travel burdens for both educators and participants. However, some participants prefer the personal interactions that in-person health programming provides [15]. After multiple requests from senior citizen centers to deliver SLEEP in person, the program was revamped to accommodate in-person instruction. These accommodations included printing out hard copies of handouts and data collection tools. All other programmatic aspects remained the same [13]. As the effectiveness of SLEEP had only been tested when delivered online, the purpose of this study was to test whether SLEEP delivery modality influenced outcomes, namely, if in-person delivery of SLEEP was superior to online delivery. Given that a recent review indicated no differences in online compared to in-person treatment for insomnia, we hypothesized that the two modalities would deliver equivalent outcomes.

## 2. Results

### 2.1. Results for the Entire Group

A total of 60 individuals participated in the SLEEP program; of these people, 6 (10%) were male, 53 (88.3%) were female, and 1 (1.7%) person chose not to answer. A breakdown of participants by race indicated that the majority of participants identified as White (*n* = 48, 80.0%), while Black/African American (*n* = 10, 16.7%) and Asian (*n* = 1, 1.7%) participants also took part. One participant did not identify their race (*n* = 1, 1.7%). The average age reported was 66.5 ± 13.2 y. The mean body mass index (BMI) was 28.7 ± 5.3 kg/m^2^. Most participants indicated that they were retired (*n* = 40, 66.7%), but others engaged in part-time (*n* = 5, 8.3%), full-time (*n* = 11, 18.3%), or some other type of employment, e.g., working and student status (*n* = 4, 6.7%). Only a small number of participants indicated having diagnosed sleep issues (*n* = 8, 13.3%).

Outcomes of interest for the whole group showed significant progress (Table 1). Sleep duration, quality, and hygiene behaviors all indicated improvement. Of note, sleep duration improved by approximately 24 min.

Before participating in SLEEP, sleep quality was associated with both sleep duration (−0.592, *p* < 0.001) and sleep hygiene practices (r = 0.311, *p* = 0.016). As sleep quality worsened, sleep duration decreased, and sleep hygiene practices worsened. Age was associated with sleep hygiene practices (r = −0.340, *p* = 0.010); as age increased, sleep hygiene practices were better. Sleep duration differed by retirement status: retired participants had a longer sleep duration compared to full-time workers (6.7 ± 1.2 vs. 5.6 ± 1.0, *p* = 0.047). Neither sleep quality nor hygiene practices differed by retirement status. No associations with BMI were observed for any of the sleep outcome variables.

After the program, better sleep quality was associated with longer sleep duration (r = −0.545, *p* < 0.001) but no longer linked to sleep hygiene practices. However, sleep hygiene practices were still associated with age (r = −0.518, *p* < 0.001), and as age increased, sleep hygiene practices improved. Retirees differed from full-time workers in terms of sleep hygiene practices after the program, engaging in a lower frequency of problematic behaviors (11.8 ± 4.3 vs. 16.9 ± 6.5, *p* = 0.024), but duration no longer differed as it did pre-program. As with baseline findings, no associations between BMI and post-program sleep outcomes were observed.

### 2.2. Results by Delivery Modality

Data were then analyzed based on delivery modality. In total, 28 (46.7%) participants received online instruction; 32 (53.3%) received SLEEP instruction in person. The number of classes attended did not differ between the groups (5.4 for online vs. 5.3 for in-person, *p* = 0.315). Groups did differ in terms of retirement composition (*p* = 0.008), with 14 retirees in the online group and 26 in the in-person group. These differences in employment status also translated to differences in age; the online group was significantly younger (59.3 ± 12.6 vs. 73.5 ± 9.6 y, *p* < 0.001). There were no differences between groups in terms of sex, race, diagnosed sleep issues, or BMI.

Differences in outcomes by delivery modality are shown in Table 2. No differences in sleep measurements were observed at baseline between the groups. The only difference between the two groups at follow-up was that the in-person group demonstrated a larger improvement in sleep hygiene practices after SLEEP compared to the in-person group.

## 3. Discussion

SLEEP successfully improved sleep duration, sleep quality, and sleep hygiene behaviors among all participants. Before the intervention, better sleep quality was associated with increased sleep duration and better sleep hygiene practices. Further, as age increased, sleep hygiene practices were better, and retired participants had a longer sleep duration compared to full-time workers. After the program, better sleep quality was also associated with longer sleep duration, but the relationship between sleep quality and sleep hygiene practices was no longer present. As with baseline data, as age increased, sleep hygiene practices improved, and retirees reported less problematic sleep hygiene practices compared to full-time workers post-program. When analyzed by delivery modality, sleep duration and sleep quality improved comparably over the course of the intervention, while sleep hygiene behaviors improved more among those in the in-person group.

These findings add to the literature supporting the effectiveness of non-expert delivered sleep programming in general, e.g., [16,17,18,19], and SLEEP in particular [13,14]. Previous testing of SLEEP at the immediate post-program timepoint demonstrated significant improvements in sleep quality and sleep hygiene behaviors compared to a control group [13]. Those improvements and an improvement in sleep duration were observed in the SLEEP cohort six months post-program as well.

While the gold standard for addressing insomnia is Cognitive Behavioral Therapy for insomnia (CBTi) [20], CBTi is not available to everyone who would benefit due to shortages of therapists, the cost of care, and accessibility issues [21,22,23]. Given that approximately one in three US adults reports failing to meet the recommended sleep duration guideline of at least 7 h per night [24], and the concerning number of nights per week U.S. adults have difficulty falling asleep (~2.5) and days feeling unrested (~3.5), it is clear that the primary care health system is not equipped to deal with the potential demand for treatment. Thus, additional methods, such as SLEEP, are needed to assist individuals with clinical and sub-clinical sleep issues as a first-line treatment option [19].

In an attempt to reduce accessibility barriers to CBTi, studies have examined the effectiveness of online delivery. Although a relatively small number of studies have directly compared online vs. in-person delivery, a recent meta-analysis of 4 studies reported that digital delivery, e.g., using a computer, internet, or smartphone, of CBTi was determined to be non-inferior to in-person treatment outcomes as measured by the Insomnia Severity Index [25]. The results of the present study are largely consistent with the findings of the meta-analysis regarding non-inferiority.

While sleep duration and quality improved similarly for the two groups, the in-person group experienced significantly greater improvements in sleep hygiene behaviors. Although baseline sleep hygiene behaviors did not differ between the in-person and online groups, age and retirement status did; in-person participants were older and more likely to be retired than online attendees. This is likely due to the fact that in-person classes were typically conducted at senior citizen centers during the workday. Upon completion of SLEEP, retirees reported less problematic sleep hygiene practices compared to full-time workers. The differences in improvement in sleep hygiene behaviors between the two groups are likely explained by the greater number of older, retired individuals in the in-person group.

While the number of retirees in the online group (*n* = 14) means that results regarding retirees should be considered with caution, age was consistently and positively associated with better sleep hygiene habits both before and after the program. While conventional wisdom suggests sleep problems are simply a part of growing older, multiple studies fail to support this idea [26,27]. Suggested possible reasons for improved sleep outcomes among older adults include having more control over their sleep schedules, e.g., fewer work or family demands on their time [27], and better attitudes towards sleep, i.e., the benefits and enjoyment of sleep and sleep as a time commitment [28]. These factors could certainly play a role in improving sleep hygiene practices.

Despite the lack of association between sleep hygiene habits and sleep outcomes post-program, sleep hygiene habits improved for the larger group. Recommendations to improve sleep hygiene to engender long-term sleep improvement remain the prevailing first-line approach to treating sleep problems [12,29]. The positive effects of sleep hygiene on sleep quality have been demonstrated in the literature [12,30,31]. However, one recent study makes an important distinction between long-term vs. acute sleep hygiene habits [32]. That study indicated relationships between sleep hygiene and sleep quality differed based on the measurement timeframe of sleep hygiene practices, with acute measures serving as a better predictor. The SHI used in the present study examines long-term habits compared to acute habits. Future work should measure both long-term and short-term changes in sleep hygiene to determine the effects on sleep outcomes of interest.

The effectiveness of sleep hygiene interventions to improve sleep duration may be more variable than their effectiveness to improve sleep quality, according to several recent reviews [19,33]. In that review, half of the programs successfully increased duration while half did not. This discrepancy likely stems from the inherent variability in the number and types of sleep hygiene behaviors targeted as well as from the variability in the populations tested. Sleep hygiene behaviors that cause sleep problems for some individuals may have no effect on others.

A sleep education curriculum delivered by non-experts that demonstrates comparable improvements in sleep duration and sleep quality, whether disseminated via in-person or online modality, is valuable to both health-related organizations and individuals. Flexibility in delivery methods allows organizations to tailor their health promotion education based on the organization’s mission, goals, resources, and clientele needs. Organizations that provide clients with a combination of tangible goods, services, and education, e.g., congregate meals and health screening, would benefit from an efficacious in-person sleep education program that facilitates the pairing of services. From the participant’s perspective, in-person programming is particularly helpful for participants who need social interaction and support, individualized instruction, increased environmental cues, and reduced environmental distractions. Additionally, an option for in-person education is necessary for individuals who have limited broadband access and/or inadequate knowledge and equipment for distance learning. Conversely, organizations that strive to promote health education by using cost-efficient strategies to reach large numbers of people might find greater benefits in the online version of SLEEP. Online delivery of SLEEP can reach clientele who travel long distances to access care, prefer the convenience of attending classes at the location of their choice, and/or have inadequate transportation, time, and/or resources, e.g., childcare and gasoline, to participate in in-person classes. Having the flexibility to choose a delivery method that best suits the organization and individual helps reduce health disparities by eliminating barriers to sleep education based on resources and/or individual preferences and needs.

Future work should be undertaken to address some of the limitations of the current study. A larger, more diverse sample should be studied to ensure the generalizability of findings across gender, race, age, and employment status. Objective measures of sleep would further strengthen findings. A randomized controlled trial would address possible issues of bias, but the goal of this study was to assess active programming adapting to real-life opportunities and constraints.

## 4. Materials and Methods

SLEEP is currently offered through Michigan State University Extension by trained SLEEP educators. Adults ages 18 years and older are eligible to enroll in the program free of charge. Participants self-enrolled in the program at a time and modality that was convenient for them, making this a quasi-experimental design. A physician’s order or sleep disorder diagnosis is not required for SLEEP. Attendees do not have to reside in the state of Michigan. Participants who enrolled in programming are invited to share their experiences as part of ongoing research to assess program effectiveness. Participants who completed the pre- and post-program surveys were included in the data analysis.

Data were collected online via Qualtrics (Provo, UT, USA) for online participants or on paper for in-person participants. Data collected included demographic and occupational status information, e.g., retired, part-time employment, etc. Height and body weight were self-reported, and body mass index (BMI) was calculated. Participants were asked if they had been diagnosed with any sleep issues such as sleep apnea, restless legs syndrome, etc.

Sleep outcomes included sleep duration, sleep quality, and sleep hygiene behaviors and were measured before and after the program. Sleep duration and quality were measured using the Pittsburgh Sleep Quality Instrument [34]. The PSQI measures sleep quality and usual sleep duration spanning the past month [34]. PSQI scores range from 0–21. A score of ≥5 indicates poor sleep quality and higher scores indicate poorer quality [34]. Sleep duration was extracted from the PSQI. Sleep hygiene practices were measured using the Sleep Hygiene Index (SHI) [35]. The SHI asks people how frequently they engage in behaviors that are not conducive to sleep health (never, rarely, sometimes, frequently, always). Examples include questions about lack of bedtime and waketime consistency, caffeine consumption, and working or doing other stressful things in the bedroom. Scores can range from 0–52.

Data were analyzed using SPSS v. 28.0.1.1 (IBM Corp, Armonk, NY, USA). Differences between groups were assessed using Student’s *t*-tests, one-way ANOVA tests with Bonferroni corrections, or chi-square tests, when appropriate. Differences within groups were evaluated using paired *t*-tests.

## 5. Conclusions

The purpose of this study was to test whether the SLEEP delivery modality, online vs. in-person, influenced programmatic outcomes. Changes in sleep duration and sleep quality did not differ between delivery methods. In-person participants reported increased improvements in sleep hygiene behaviors compared to online participants, but sleep hygiene behaviors were not associated with changes in sleep duration or quality. Based on these findings, SLEEP appears to be equally effective when delivered online or in person.

## Figures and Tables

**Table 1:** Sleep outcomes before and after SLEEP for the entire sample (*n* = 60).

	Pre	Post	*p*-Value
Sleep duration (h)	6.4 ± 1.2	6.8 ± 1.0	<0.001
Sleep quality (PSQI)	7.6 ± 3.1	5.7 ± 2.5	<0.001
Sleep hygiene (SHI)	16.4 ± 7.1	13.5 ± 5.4	<0.001

Data in the table provide mean ± standard deviation information and *p*-values for comparisons between the two timepoints for each outcome. All outcomes improved after completion of SLEEP. Sleep duration data were extracted from the Pittsburg Sleep Quality Index (PSQI). PSQI data indicate PSQI scores. Sleep hygiene data reflect scores on the Sleep Hygiene Index (SHI). Lower scores on the PSQI and SHI indicate improved outcomes.

**Table 2:** Differences in outcomes between online and in-person delivery.

	Online (*n* = 28)	In-Person (*n* = 32)	*p*-Value
Sleep duration pre (h)	6.5 ± 1.2	6.3 ± 1.3	0.521
Sleep duration post (h)	7.0 ± 1.0	6.7 ± 1.0	0.274
Sleep quality pre (PSQI)	7.1 ± 2.9	8.1 ± 3.1	0.215
Sleep quality post (PSQI)	5.4 ± 2.6	6.0 ± 2.5	0.357
Sleep hygiene pre (SHI)	16.7 ± 5.7	16.0 ± 8.2	0.631
Sleep hygiene post (SHI)	15.1 ± 5.4	12.1 ± 5.2	0.033 *

Data in the table provide mean ± standard deviation information and *p*-values for comparisons between the two groups for each outcome. Participants in the in-person group demonstrated a significant improvement compared to participants in the online group for sleep hygiene behaviors at the end of the program. Sleep duration data were extracted from the Pittsburg Sleep Quality Index (PSQI). PSQI data indicate PSQI scores. Sleep hygiene data reflect scores on the Sleep Hygiene Index (SHI). Lower scores on the PSQI and SHI indicate improved outcomes. * indicates a significant difference between the groups.

## Data Availability

Data are still undergoing analysis. Requests for data can be made from the corresponding author.
